# Pregnancy by Assisted Reproductive Technology Is Associated with Shorter Telomere Length in Neonates

**DOI:** 10.3390/ijms21249688

**Published:** 2020-12-18

**Authors:** Toshiko Minamoto, Kentaro Nakayama, Tomoka Ishibashi, Masako Ishikawa, Kohei Nakamura, Hitomi Yamashita, Kamrunnahar Shanta, Hossain Mohammad Mahmud, Sultana Razia, Kouji Iida, Gyosuke Sakashita, Tsukasa Nakamura, Hideyuki Kanda, Satoru Kyo

**Affiliations:** 1 Department of Obstetrics and Gynecology, Shimane University School of Medicine, Izumo 693-8501, Japan; minamoto@med.shimane-u.ac.jp (T.M.); tomoka@med.shimane-u.ac.jp (T.I.); m-ishi@med.shimane-u.ac.jp (M.I.); kohei320@med.shimane-u.ac.jp (K.N.); memedasudasu1103@gmail.com (H.Y.); kamrunnahar.vet@gmail.com (K.S.); likhon.vet@gmail.com (H.M.M.); raeedahmed@yahoo.com (S.R.); iida@med.shimane-u.ac.jp (K.I.); satoruky@med.shimane-u.ac.jp (S.K.); 2 Department of Biochemistry, Shimane University School of Medicine, Izumo 693-8501, Japan; gsakashi@med.shimane-u.ac.jp; 3 Department of Infectious Disease, Shimane Prefectural Central Hospital, Izumo 693-8555, Japan; tsukanam@spch.izumo.shimane.jp; 4 Department of Public Health, Okayama University Graduate School of Medicine, Dentistry and Pharmaceutical Sciences, Okayama 700-8558, Japan; hkanda@okayama-u.ac.jp

**Keywords:** assisted reproductive technology, developmental origins of health and disease, pregnancy, lifestyle, neonates, telomere length

## Abstract

Telomere length (TL) influences the development of lifestyle-related diseases, and neonatal TL may influence their prevalence. Various factors have been reported to affect neonatal TL. Although the fetus is exposed to multiple conditions in utero, the main factors affecting the shortening of neonatal TL are still not known. In this study, we sought to identify factors that influence fetal TL. A total of 578 mother-newborn pairs were included for TL analysis. TL was measured in genomic DNA extracted from cord blood samples using quantitative PCR. The clinical factors examined at enrollment included the following intrauterine environmental factors: maternal age, assisted reproductive technology (ART) used, body mass index (BMI), gestational diabetes mellitus (GDM), maternal stress, smoking, alcohol consumption, preterm delivery, small-for-gestational-age, neonatal sex, and placental weight. Univariate and multivariate regression analyses were used to verify the relationship between neonatal TL and these clinical factors. The median neonatal TL to single-copy gene ratio was 1.0. Pregnancy with ART was among the 11 factors associated with shorter neonatal TL. From multiple regression analysis, we determined that neonatal TL was significantly shorter for pregnancies in the ART group than in the other groups. We conclude that pregnancy with ART is associated with shorter neonatal TL.

## 1. Introduction

The general idea of the “Developmental Origins of Health and Disease” hypothesis, in which various environmental factors influence postnatal development and the risk of disease onset during adulthood, is intriguing [1]. The concept is that future health and susceptibility to certain illnesses are strongly influenced by the prenatal and early postnatal environments. Barker et al. reported that low birth weight infants have an increased risk of developing metabolic diseases in adulthood, such as diabetes, hypertension, and hyperlipidemia, and they proposed the “Fetal Origins of Adult Disease” hypothesis [2]. Furthermore, Gluckman and Hanson developed the Developmental Origins of Health and Disease hypothesis [3]. According to this hypothesis, exposure to various environments during development causes a predictive adaptive response, and future disease risk is determined by the degree of adaptation to subsequent environments.

In addition, preemptive medicine, which provides early intervention for disease risk based on individual genetic backgrounds, has attracted increasing attention. In this context, it is important to focus on fetal factors that can influence neonatal telomere length (TL), which may be relevant for the development of cardiovascular diseases, diabetes, and malignancies in adults [4,5,6].

Telomeres are located at the ends of chromosomes and consist of a characteristic DNA sequence (TTAGGG), along with various proteins. Telomeres play an important role in preserving chromosomal stability [7] and become shorter after each round of cell division. Considering that a strong correlation has been identified between TL and lifespan, telomeres have attracted attention as biomarkers for aging [8,9]. TL is uniformly shortened during aging within the same organism, but differs between individuals. In addition, Okuda et al. reported that the variability in TL observed between adults reflects fetal differences in TL [10].

More recently, an association between maternal perinatal factors, fetal umbilical blood, and placental TL was reported in several unrelated studies [11]. It is believed that individual differences in TL may be determined by the intrauterine environment, which is in turn affected by stress, diabetes, obesity, smoking, and small-for-gestational-age status [11,12,13,14,15,16,17]. Thus, maternal health conditions and lifestyle habits may exert a significant influence on fetal programming, which could affect health in adulthood. Although some pregnant women have many of these risk factors, the factors that have a strong effect on TL have yet to be investigated. Therefore, in this study, we sought to define the major determinants of fetal TL.

## 2. Results

A total of 578 mother-newborn pairs were selected for TL analysis using quantitative PCR. We confirmed the correlation between the T/S ratio and TRF in 12 randomly selected umbilical cord blood samples. A strong positive correlation has been found between the T/S ratio assessment by q-PCR and TRF measurement as determined by Southern blotting (Supplementary Figure S1). The distribution of infants’ TL is shown in Supplementary Figure S2. The median neonatal TL to single-copy gene (T/S) ratio in cord blood was 1.0 (interquartile range, 0.7–1.5). Demographic characteristics and perinatal factors are listed in Table 1. The mean age of the pregnant women was 31.6 ± 5 (range, 17–46) years. Of the 578 pregnant women, 175 (30%) were older than 35 years. There were 58 women (10%) who became pregnant via assisted reproductive technology (ART), and 28 of these women used conventional in vitro fertilization, whereas 30 became pregnant by intracytoplasmic sperm injection. Mean body mass index (BMI) at 12 weeks was 21.3 kg/m^2^. In terms of body weight, 97 women (17%) were underweight, 408 (71%) had standard weight, and 73 (13%) were obese. Forty-two women (7.3%) had gestational diabetes mellitus (GDM), 181 (31%) displayed a stress score ≥300 (i.e., high stress), 35 (6.1%) smoked throughout pregnancy, and 38 (6.7%) drank alcohol during pregnancy.

Among the 578 newborns, 298 were male (52%); the mean gestational age was 38.6 (range, 29–42) weeks, and mean (±standard deviation) birth weight was 2962 ± 373.7 g. Thirty-eight newborns (6.7%) were preterm, 41 (7%) were small-for-gestational-age, and 187 (32.4%) had a small placenta.

Univariate correlation analysis of the relationship between TL and each perinatal factor is shown in Table 2. ART was among the 11 factors associated with shorter TL in cord blood. Maternal obesity also tended to be associated with shorter TL in DNA extracted from cord blood. Multiple regression analysis showed that the TL of DNA in neonatal cord blood was shorter in the ART group (*p* = 0.013). Obesity during early pregnancy was not significantly associated with shorter TL (Table 3). Furthermore, in the group that achieved pregnancy via ART, there was no significant difference in TL between women treated with intracytoplasmic sperm injection and those who underwent conventional in vitro fertilization. In addition, the average age of the ART group was 34.9 years and that of the non-ART group was 31.3 years; the individuals in the ART group tended to be older, but the difference in age between these groups was not significant.

## 3. Discussion

In recent years, preemptive medical care has drastically changed the therapeutic approach to various diseases. In this respect, the fetal environment offers a wide scope for various extensive investigations. Telomeres are known to be good biomarkers for determining lifetime disease risk. Diseases related to shorter telomeres include heart disease, pulmonary fibrosis, dementia, loss of immune function, diabetes, arteriosclerosis, malignancies, and renal disease [8,18,19,20,21,22,23,24,25]. Therefore, we focused on TL as a biomarker associated with the effects of intrauterine factors. According to recent studies, maternal factors related to the shortening of TL in DNA extracted from cord blood include maternal obesity, high stress, diabetes, smoking, sleeping time, folate levels, neonatal sex, and maternal education [11,12,13,14,15,16,17,26,27,28,29]. Low birth weight and sex have been reported as factors influencing TL in newborns. Male neonates have shorter TL in DNA extracted from cord blood [17,30]. The impact of single factors on telomere shortening is an interesting subject of research. However, because pregnant women may be exposed to multiple risk factors simultaneously, we analyzed the correlations between several factors and neonatal TL.

ART was identified as a factor associated with shorter neonatal TL using multivariate analysis. Among the various perinatal factors, ART may be strongly associated with neonatal TL. As delivery after ART was recently found to be associated with various diseases, we included it as a factor in our analysis [31,32,33]. Maternal obesity at 12 weeks of gestation also tended to be associated with TL shortening, but a significant correlation was not observed in multiple regression analysis.

This is the first report to demonstrate that pregnancies with ART are associated with neonatal telomere shortening. Scherrer et al. reported that vascular endothelial function in newborns from ART-induced pregnancies is reduced compared with that in newborns from natural pregnancies [32]. Furthermore, Meister et al. reported that the incidence of hypertension is significantly increased during adolescence in individuals who were conceived by ART [31]. Thus, based on previous reports and our present findings, the prognosis of children conceived by ART should be investigated carefully.

Telomeres are located at the ends of chromosomes and play a role as substrates in chromosome protection and replication. Extreme telomere shortening causes DNA damage and vascular endothelial aging, induces cellular senescence as a consequence of activation of the p53 pathway, and is involved in the pathogenesis of cardiovascular diseases [23]. Telomere shortening may result from oxidative stress, which is generated during ART procedures [34,35,36]. Agarwal et al. reported that oxidative stress occurs at various stages in the ART process, causing DNA damage [37]. We could not confirm the detailed conditions for ART in our survey, and it may be necessary to explore this in the future. Our findings suggest the need for the long-term investigation of children conceived by ART and for the examination of the correlation between oxidative stress and TL when using ART. The extent of oxidative stress generated when using ART, as well as its impact on TL in fertilized embryos and fetuses, should be investigated. In our study, there was no significant difference in TL between the different types of ART used. However, more data should be obtained by increasing the number of subjects. Other ART factors that could be related to telomere shortening may need to be investigated, such as fertilized egg quality, patient’s medical health status, and the effects of cytokines in the pelvis on infertility. In addition, a prospective study aimed at confirming whether ART is involved in the shortening of newborn telomeres is needed in the future.

Previously reported factors in TL shortening (maternal age, maternal obesity in early pregnancy, diabetes, high-stress status, smoking, alcohol consumption, preterm birth, intrauterine growth retardation, and neonatal sex) did not have significant effects on TL shortening in the present study. Recent reports have noted correlations of TL with paternal socioeconomic status and age [38,39], but we did not include these factors in this study because we sought to focus on the effects of maternal perinatal factors in the uterine environment. Univariate analysis revealed a marginally significant effect of maternal obesity in early pregnancy, consistent with previous reports [15]. However, all previously published studies on maternal obesity were conducted in Western countries on women with a significantly higher degree of obesity than in our sample population. Most of the obese Japanese pregnant women included in the present study had a BMI in the range of 25–30, and no significant correlation was found between BMI and neonatal TL. In Japanese women, the severity of obesity is not sufficiently high to cause TL shortening in newborns. Ethnicity may be one possible reason for the discrepancies between the results of this and previous studies. Although the homogeneity of the study population was one of the strengths of this study, the inclusion of only Japanese women was also one of its limitations. Another potential limitation of our study is DNA quality, which may affect the results of quantitative PCR. In the current study, we identified six cases with a very low T/S ratio, which may have been caused by the quality of the purified DNA.

Many pregnant women experience stress due to changes in eating habits and sleeping patterns, relationship difficulties, and serious problems such as domestic abuse and depression. It may have been difficult to assess maternal stress from the score obtained using the Holmes and Rahe Stress Scale questionnaire alone.

In our study, we included many women with mild pregnancy-induced diabetes and a few women with serious uncontrolled diabetes. This might be the reason why maternal diabetes did not show a significant correlation with neonatal TL. Premature birth and intrauterine growth retardation and their effect on TL remain controversial [17,40,41,42], but these factors did not show significant effects in this study. Although our results do not rule out the overall importance of previously identified factors in TL shortening, the severity and relevance of these factors might be lower in the Japanese population than in Western populations.

## 4. Experimental Section

### 4.1. Study Design, Patient Selection, and Clinical Information

This study was conducted in a retrospective manner. The study protocol was approved by the Ethics Committee of Shimane University Hospital (approval no. 2020419-1) and performed in accordance with the Declaration of Helsinki. We focused on singleton pregnancies delivered at Shimane University Hospital between May 2016 and May 2018, and written informed consent for the analysis of neonatal TL and maternal perinatal factors was obtained from all participants. Eligible women were between 17 and 46 years of age. Among the 848 mother-newborn pairs examined in this period, 578 (68.1%) were ultimately included in the analyses; the remaining were excluded because of multiple births, fetal anomalies, poor sampling, poor DNA extraction, or missing data. All mother-newborn pairs were Japanese. Our previous study subsample comprised subjects in whom maternal age, BMI, GDM, smoking, alcohol consumption, preterm birth, gestational age, birth weight, fetal growth restriction, neonatal sex, and TL in cord blood samples were determined. In the present study, additional measures of maternal stress, ART, and placental weight were examined. Informed consent for the new analyses was obtained in the form of an opt-out on the registration website. Those who opted out were excluded.

Eleven factors were analyzed for their association with neonatal TL: maternal age, ART, obesity, GDM, maternal stress, smoking, alcohol consumption, preterm delivery, small-for-gestational-age, neonatal sex, and placenta weight. We selected perinatal factors for telomere shortening with reference to previous literature. There is no report found until now on the relationship between ART and neonatal TL, but Meister et al. reported the presence of hypertension in adolescents conceived by ART [33], so we investigated if there was a correlation. Women who were 35 years of age or older were classified as elderly pregnant women. In 1958, the International Federation of Gynecology and Obstetrics defined primiparas aged over 35 years as elderly pregnancies; therefore, we used 35 years as a cut-off point. The degree of obesity was divided into three categories based on BMI: emaciated (BMI < 18.5), standard (18.5 < BMI ≤ 25), and obese (BMI ≥ 25), according to the classification assigned by the World Health Organization [43]. Martens et al. reported that obesity was associated with neonatal TL [15]. Maternal GDM has been previously linked to fetal TL shortening [14]. GDM in pregnant women was diagnosed during pregnancy. Data on conditions, such as diabetes mellitus, GDM, stress, exposure to smoking (habitual active or passive smoking), and alcohol consumption during pregnancy were obtained from medical records. Maternal psychosocial stress was assessed using the Holmes and Rahe Stress Scale questionnaire [44], and subjects with 300 points or more were included in the high-stress group. This classification was based on previous evidence indicating that the persistence of such high scores for more than 1 year is associated with higher disease risk [45]. Preterm birth was defined as delivery at less than 37 weeks of gestation. Data on gestational age, placental and birth weight, fetal growth restriction, and neonatal sex were obtained from medical records. Although there have been few reports on neonatal TL in preterm infants, such infants have short telomeres [46], and fetal growth restriction is associated with shortened telomeres, although this finding is controversial. The criteria for small-for-gestational-age were defined according to the standards established by the Japanese Society of Pediatrics [47]. In addition, because TL is clearly shorter in men than in women [48], neonatal sex was added to the study items. A small placenta was defined as a placenta weighing less than 500 g.

### 4.2. Genomic DNA Extraction

Umbilical cord blood was drawn within 10 min after delivery and stored in plastic whole blood tubes spray-coated with K_2_EDTA. Peripheral blood mononuclear cells were isolated from whole blood using Lymphoprep (Alere Technologies AS, Oslo, Norway) by centrifugation at 1300× *g* for 30 min for 5 min and stored at −20 °C. Genomic DNA was isolated using a DNeasy Blood and Tissue Kit (QIAGEN, Hilden Germany) and stored at −20 °C until analysis. DNA quantity and purity were assessed by a NanoDrop spectrophotometer (Thermo Scientific, Waltham, MA, USA).

### 4.3. Southern Blotting

The mean length of the telomere restriction fragment (TRF) was measured by partially modifying the method described by Slagboom et al. [49]. Briefly, purified genomic DNA was digested by an optimized mixture of frequently cutting restriction enzymes (Hinf I: Rsa I=1:1) at 37 °C for 2 h. The sequence specificity of these enzymes ensures that telomeric and subtelomeric DNA is not cut, while non-telomeric-DNA is digested to low molecular weight fragments. Following DNA digestion, the DNA fragments were separated by gel electrophoresis and transferred to a nylon membrane by Southern blotting. The blotted DNA fragments were hybridized to a digoxigenin (DIG)-labeled probe specific for telomeric repeats and incubated with a DIG-specific antibody covalently coupled to alkaline phosphate. Finally, antibody-conjugated alkaline phosphatase was used to metabolize CDP-Star, a highly sensitive chemiluminescent substrate. This produced a visible signal that indicated the location of the immobilized telomere probe (and, hence, that of the TRF) on the blot.

### 4.4. Quantitative PCR

The method described by Cawthon et al. [50] was used to quantify sample DNA levels based on the cycle threshold (2^−ΔΔCT^) values obtained by quantitative real-time PCR. Before starting the experiment, we confirmed the correlation between the T/S ratio and telomeric restriction fragment length in 12 randomly selected neonatal samples (Supplementary Figure S1). The reactions were performed in triplicate using a Thermal Cycler D (Takara Bio, Kusatsu, Japan). The T/S ratio was determined from 35 ng DNA with Fast Start Universal SYBR Green Master (Rox). The primer sequences were: tel1, 5′-GGTTTTTGAGGGTGAGGGTGAGGGTGAGGGTGAGGGT-3′; tel2, 5′-TCCCGACTATCCCTATCCCTATCCCTATCCCTATCCCTA-3′; 36B4u, 5′-CAGCAAGTGGGAAGGTGTAATCC-3′; and 36B4d, 5′-CCCATTCTATCATCAACGGGTACAA-3′. The primer concentrations were 270, 900, 300, and 500 nM for tel1, tel2, 36B4u, and 36B4d, respectively. Hot start activation for 2 min at 95 °C was performed for single-copy gene and telomere amplification, followed by 40 cycles of 15 s at 95 °C (denaturation) and 2 min at 54 °C (annealing/extension). Triplicates of telomere runs showed a coefficient of variation (CV) of 0.62%, those of single-copy gene runs showed a CV of 0.65%, and those of T/S ratios showed a CV of 6.0% for cord blood.

### 4.5. Statistical Analysis

As the distribution of TL is biased, we performed base-10 logarithmic transformation on TL. For continuous variables, to facilitate the clinical use of our data, conversion to binary variables was also performed. For all factors, the data were analyzed using descriptive statistics. Log_10_ TL univariate analysis was performed using regression analysis for all factors. Multivariate regression analysis was performed for the relationship between TL and the 11 clinically important factors (ART, BMI, GDM, maternal stress, smoking, alcohol consumption, maternal age, preterm birth, small-for-gestational-age, neonatal sex, and placental weight). All statistical analyses were conducted using Stata 15 (SPSS, Inc., Cary, NC, USA), with the threshold for statistical significance set at *p* < 0.05.

## 5. Conclusions

Pregnancy by ART is associated with shorter TL in Japanese neonates. Given that pregnancy by ART is expected to continue increasing worldwide, the results of this study might be relevant to a new generation of human beings conceived by ART who could develop lifestyle-related diseases later in life. Therefore, further research in this field is urgently needed.

## Figures and Tables

**Table 1:** Characteristics of the participants (N = 578).

	*n*	%	M	SD
Maternal factor				
Age (years)			31.6	5
ART	58	10		
BMI (kg/m^2^)			21.3	3.9
BMI < 18.5	97	16.8	17.5	0.8
18.5 ≤ BMI < 25	408	70.6	20.8	1.6
BMI ≥ 25	73	12.6	29.08	3.8
GDM	42	7.3		
Maternal stress exposure during pregnancy (Holmes and Rahe Stress Scale questionnaire)			258.5	157.2
Holmes and Rahe Stress Scale ≥ 300	181	31.3		
Smoking	35	6.1		
Drinking	38	6.6		
Gestational age (weeks)			38.6	1.6
Preterm birth	38	6.6		
Birth weight (g)			2962	373.7
SGA	30	5.2		
Newborn sex (male)	298	51.6		
Placental weight (g)			543.2	107.9
Placental weight < 500 g	187	32.4		
Cord blood telomere length (T/S ratio) ^a^			1.0 ^b^	0.7–1.5 ^c^

Note. SD = standard deviation; M = mean; ART = assisted reproductive technology; BMI = body mass index; ICSI = intracytoplasmic sperm injection; GDM = gestational diabetes mellitus; SGA = small for gestational age; T/S ratio = ratio of telomere repeats copy number to single-gene copy number. ^a^ Log-transformed to obtain normal distributions. ^b^ Median. ^c^ Interquartile range.

**Table 2:** Correlation between telomere length and each perinatal factor.

Variables	All (*n* = 578) No. (% of All)	Telomere Length M	*p*-Value
Maternal factor			
Age (years)			
≥35	175 (30)	1.02	
< 35	403 (70)	0.99	0.543
ART			
Yes	58 (10)	0.80	
No	520 (90)	1.04	**0.003**
BMI			
≥25	73 (13)	0.88	
<25	505 (87)	1.03	0.057
GDM			
Yes	42 (7.3)	0.99	
No	536 (92.7)	1.01	0.784
Stress			
≥300	181 (31)	1.02	
<300	397 (69)	1.01	0.907
Smoking status			
Yes	35 (6.1)	1.04	
No	543 (93.9)	1.01	0.764
Alcohol consumption			
Yes	38 (6.7)	1.12	
No	540 (93.3)	1.01	0.321
Newborn factor			
Preterm birth			
Yes	38 (6.7)	1.07	
No	540 (93.3)	1.01	0.596
SGA			
Yes	30 (5.2)	0.96	
No	548 (94.8)	1.01	0.632
Sex			
Male	298 (52)	0.99	
Female	280 (48)	1.01	0.869
Placenta weight			
<500 g	187 (32)	1.01	
≥500 g	391 (68)	1.01	0.945

Note. Abbreviations used in this table are the same as in Table 1. Bold indicates statistical significance (*p* < 0.05).

**Table 3:** Multivariate correlation analysis of telomere length with material perinatal factors.

Variables	Coefficient	95% Confidence Interval		*p*-Value
Age ≥ 35 years	−0.0051	−0.0559	0.0457	0.843
ART	−0.0967	−0.1733	−0.0201	**0.013**
BMI ≥ 25	−0.0494	−0.1192	0.0204	0.165
GDM	0.0009	−0.0875	0.0892	0.985
Stress ≥ 300	0.0005	−0.0481	0.0491	0.984
Smoking	0.0026	−0.0934	0.0987	0.957
Alcohol	0.0393	−0.0530	0.1317	0.403
Preterm delivery	0.0438	−0.0526	0.1403	0.372
Fetal growth restriction	−0.0228	−0.1322	0.0865	0.682
Male sex	−0.0069	−0.0524	0.0385	0.764
Placental weight < 500 g	0.0001	−0.0001	0.0003	0.347

Note. Abbreviations used in this table are the same as in Table 1. Bold indicates statistical significance (*p* < 0.05).

## Supplementary Materials

The following are available online at https://www.mdpi.com/1422-0067/21/24/9688/s1.

## References

[1] Gluckman P.D., Hanson M.A., Beedle A.S. (2007). Early life events and their consequences for later disease: A life history and evolutionary perspective. Am. J. Hum. Biol..

[2] Barker D.J., Hanson M.A. (1990). The fetal and infant origins of adult disease. BMJ.

[3] Gluckman P.D. (2004). Living with the past: Evolution, development, and patterns of disease. Science.

[4] Fitzpatrick A.L., Kronmal R.A., Gardner J.P., Psaty B.M., Jenny N.S., Tracy R.P., Walston J., Kimura M., Aviv A. (2007). Leukocyte telomere length and cardiovascular disease in the cardiovascular health study. Am. J. Epidemiol..

[5] Sampson M., Winterbone M.S., Hughes J.C., Dozio N., Hughes D.A. (2006). Monocyte telomere shortening and oxidative DNA damage in type 2 diabetes. Diabetes Care.

[6] Ma H., Zhou Z., Wei S., Liu Z., Pooley K.A., Dunning A.M., Svenson U., Roos G., Hosgood H.D., Shen M. (2011). Shortened telomere length is associated with increased risk of cancer: A meta-analysis. PLoS ONE.

[7] Yao M.C., Blackburn E., Gall J. (1981). Tandemly repeated C-C-C-C-A-A hexanucleotide of Tetrahymena rDNA is present elsewhere in the genome and may be related to the alteration of the somatic genome. J. Cell Biol..

[8] Haycock P.C., Heydon E.E., Kaptoge S., Butterworth A.S., Thompson A., Willeit P. (2014). Leucocyte telomere length and risk of cardiovascular disease: Systematic review and meta-analysis. BMJ.

[9] Brouilette S.W., Moore J.S., McMahon A.D., Thompson J.R., Ford I., Shepherd J., Packard C.J., Samani N.J. (2007). Telomere length, risk of coronary heart disease, and statin treatment in the West of Scotland Primary Prevention Study: A nested case-control study. Lancet.

[10] Okuda K., Bardeguez A., Gardner J.P., Rodriguez P., Ganesh V., Kimura M., Skurnick J., Awad G., Aviv A. (2002). Telomere length in the newborn. Pediatr. Res..

[11] Whiteman V.E., Goswami A., Salihu H.M. (2017). Telomere length and fetal programming: A review of recent scientific advances. Am. J. Reprod. Immunol..

[12] Youngren K., Jeanclos E., Aviv H., Kimura M., Stock J., Hanna M., Skurnick J., Bardeguez A., Aviv A. (1998). Synchrony in telomere length of the human fetus. Hum. Genet..

[13] Oliveira B.S., Zunzunegui M.V., Quinlan J., Fahmi H., Tu M.T., Guerra R.O. (2016). Systematic review of the association between chronic social stress and telomere length: A life course perspective. Ageing Res. Rev..

[14] Biron-Shental T., Sukenik-Halevy R., Naboani H., Liberman M., Kats R., Amiel A. (2015). Telomeres are shorter in placentas from pregnancies with uncontrolled diabetes. Placenta.

[15] Martens D.S., Plusquin M., Gyselaers W., De Vivo I., Nawrot T.S. (2016). Maternal pre-pregnancy body mass index and newborn telomere length. BMC Med..

[16] Salihu H.M., Pradhan A., King L., Paothong A., Nwoga C., Marty P.J., Whiteman V. (2015). Impact of intrauterine tobacco exposure on fetal telomere length. Am. J. Obstet. Gynecol..

[17] Akkad A., Hastings R., Konje J.C., Bell S.C., Thurston H., Williams B. (2006). Telomere length in small-for-gestational-age babies. BJOG Int. J. Obstet. Gynaecol..

[18] Codd V., Nelson C.P., Albrecht E., Mangino M., Deelen J., Buxton J.L., Hottenga J.J., Fischer K., Esko T., Surakka I. (2013). Identification of seven loci affecting mean telomere length and their association with disease. Nat. Genet..

[19] Alder J.K., Hanumanthu V.S., Strong M.A., DeZern A.E., Stanley S.E., Takemoto C.M., Danilova L., Applegate C.D., Bolton S.G., Mohr D.W. (2018). Diagnostic utility of telomere length testing in a hospital-based setting. Proc. Natl. Acad. Sci. USA.

[20] Yaffe K., Lindquist K., Kluse M., Cawthon R., Harris T., Hsueh W.-C., Simonsick E.M., Kuller L., Li R., Ayonayon H.N. (2011). Telomere length and cognitive function in community-dwelling elders: Findings from the Health ABC Study. Neurobiol. Aging.

[21] Kaszubowska L. (2008). Telomere shortening and ageing of the immune system. J. Physiol. Pharmacol..

[22] Willeit P., Raschenberger J., Heydon E.E., Tsimikas S., Haun M., Mayr A., Weger S., Witztum J.L., Butterworth A.S., Willeit J. (2014). Leucocyte telomere length and risk of type 2 diabetes mellitus: New prospective cohort study and literature-based meta-analysis. PLoS ONE.

[23] Minamino T., Komuro I. (2008). Vascular aging: Insights from studies on cellular senescence, stem cell aging, and progeroid syndromes. Nat. Clin. Pract. Neurol..

[24] Willeit P., Willeit J., Mayr A., Weger S., Oberhollenzer F., Brandstätter A., Kronenberg F., Kiechl S. (2010). Telomere length and risk of incident cancer and cancer mortality. JAMA.

[25] Mazidi M., Rezaie P., Covic A., Cheung M., Rysz J., Kengne A.P., Banach M. (2017). Telomere attrition, kidney function, and prevalent chronic kidney disease in the United States. Oncotarget.

[26] Entringer S., Epel E.S., Lin J., Buss C., Shahbaba B., Blackburn E.H., Simhan H.N., Wadhwa P.D. (2013). Maternal psychosocial stress during pregnancy is associated with newborn leukocyte telomere length. Am. J. Obstet. Gynecol..

[27] Marchetto N.M., Glynn R.A., Ferry M.L., Ostojic M., Wolff S.M., Yao R., Haussmann M.F. (2016). Prenatal stress and newborn telomere length. Am. J. Obstet. Gynecol..

[28] Salihu H.M., King L., Patel P., Paothong A., Pradhan A., Louis J., Naik E., Marty P.J., Whiteman V. (2015). Association between maternal symptoms of sleep disordered breathing and fetal telomere length. Sleep.

[29] Louis-Jacques A., Salihu H.M., King L.M., Paothong A., Sinkey R.G., Pradhan A., Riggs B.M., Siegel E.M., Salemi J., Whiteman V.E. (2016). A positive association between umbilical cord RBC folate and fetal TL at birth supports a potential for fetal reprogramming. Nutr. Res..

[30] Wojcicki J.M., Olveda R., Heyman M.B., Elwan D., Lin J., Blackburn E.H., Epel E.S. (2016). Cord blood telomere length in Latino infants: Relation with maternal education and infant sex. J. Perinatol..

[31] Meister T.A., Rimoldi S.F., Soria R., von Arx R., Messerli F.H., Sartori C., Scherrer U., Rexhaj E. (2018). Association of assisted reproductive technologies with arterial hypertension during adolescence. J. Am. Coll. Cardiol..

[32] Scherrer U., Rimoldi S.F., Rexhaj E., Stuber T., Duplain H., Garcin S., De Marchi S.F., Nicod P., Germond M., Allemann Y. (2012). Systemic and pulmonary vascular dysfunction in children conceived by assisted reproductive technologies. Circulation.

[33] Chen M., Heilbronn L.K. (2017). The health outcomes of human offspring conceived by assisted reproductive technologies (ART). J. Dev. Orig. Health Dis..

[34] Agarwal A., Aponte-Mellado A., Premkumar B.J., Shaman A., Gupta S. (2012). The effects of oxidative stress on female reproduction: A review. Reprod. Biol. Endocrinol..

[35] Von Zglinicki T. (2002). Oxidative stress shortens telomeres. Trends Biochem. Sci..

[36] Bedaiwy M.A., Falcone T., Mohamed M.S., Aleem A.A., Sharma R.K., Worley S.E., Thornton J., Agarwal A. (2004). Differential growth of human embryos in vitro: Role of reactive oxygen species. Fertil. Steril..

[37] Agarwal A., Saleh R.A., Bedaiwy M.A. (2003). Role of reactive oxygen species in the pathophysiology of human reproduction. Fertil. Steril..

[38] Martens D.S., Janssen B.G., Bijnens E.M., Clemente D.B.P., Vineis P., Plusquin M., Nawrot T.S. (2020). Association of Parental Socioeconomic Status and Newborn Telomere Length. JAMA Netw. Open.

[39] Heidinger B., Young R.C. (2020). Cross-Generational effects of parental age on offspring longevity: Are telomeres an important underlying mechanism?. BioEssays.

[40] Turner K.J., Vasu V., Greenall J., Griffin D.K. (2014). Telomere length analysis and preterm infant health: The importance of assay design in the search for novel biomarkers. Biomark. Med..

[41] Menon R., Yu J., Basanta-Henry P., Brou L., Berga S.L., Fortunato S.J., Taylor R.N. (2012). Short Fetal Leukocyte Telomere Length and Preterm Prelabor Rupture of the Membranes. PLoS ONE.

[42] Davy P., Nagata M., Bullard P., Fogelson N., Allsopp R. (2009). Fetal Growth Restriction is Associated with Accelerated Telomere Shortening and Increased Expression of Cell Senescence Markers in the Placenta. Placenta.

[43] World Health Organization, WHO Expert Consultation (2004). Appropriate body-mass index for Asian populations and its implications for policy and intervention strategies. Lancet.

[44] Homas T.H., Richard H.R. (1967). The social readjustment rating scale. J. Psychosom. Res..

[45] Judith G.R., Elmer L. (1976). Life Events, Stress, and Illness Their nature and effects. Science.

[46] Friedrich U., Schwab M., Griese E.-U., Fritz P., Klotz U. (2001). Telomeres in Neonates: New Insights in Fetal Hematopoiesis. Pediatr. Res..

[47] Introduction of new gestational age standard values at birth. https://www.jpeds.or.jp/uploads/files/saisin_100826.pdf.

[48] Brown L., Needham B., Ailshire J. (2017). Telomere Length Among Older U.S. Adults: Differences by Race/Ethnicity, Gender, and Age. J. Aging Health.

[49] Slagboom P.E., Droog S., Boomsma D.I. (1994). Genetic determination of telomere size in humans: A twin study of three age groups. Am. J. Hum. Genet..

[50] Cawthon R.M. (2002). Telomere measurement by quantitative PCR. Nucleic Acids Res..

