# Supplementary material for: Pregnancy by Assisted Reproductive Technology Is Associated with Shorter Telomere Length in Neonates
Source: Int J Mol Sci. 2020 Dec 18;21(24):9688. doi: 10.3390/ijms21249688 (PMC7766074; doi:10.3390/ijms21249688)
Supplement: Supplementary file 1 [file ijms-21-09688-s001.pdf]

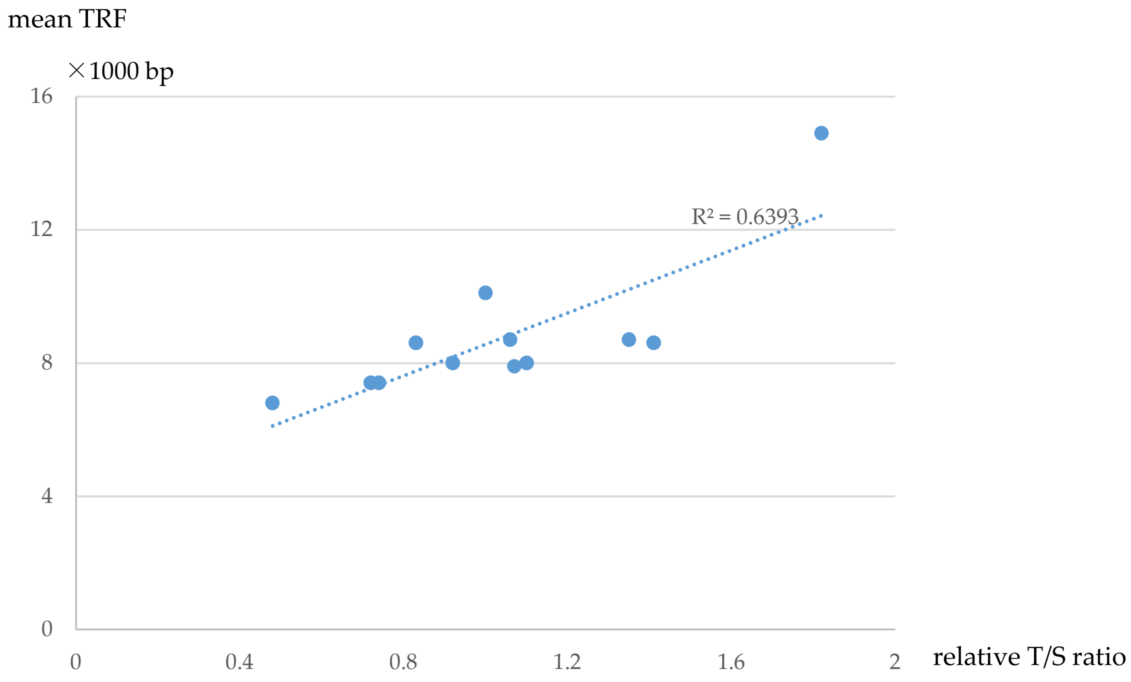

Correlation between the T/S ratio and TRF

Figure. S1. Correlation between the relative telomere length to single-copy gene (T/S) ratio determined by quantitative PCR and mean telomeric restriction fragment (TRF) length determined by Southern blot analysis using DNA samples prepared from neonatal cord blood.

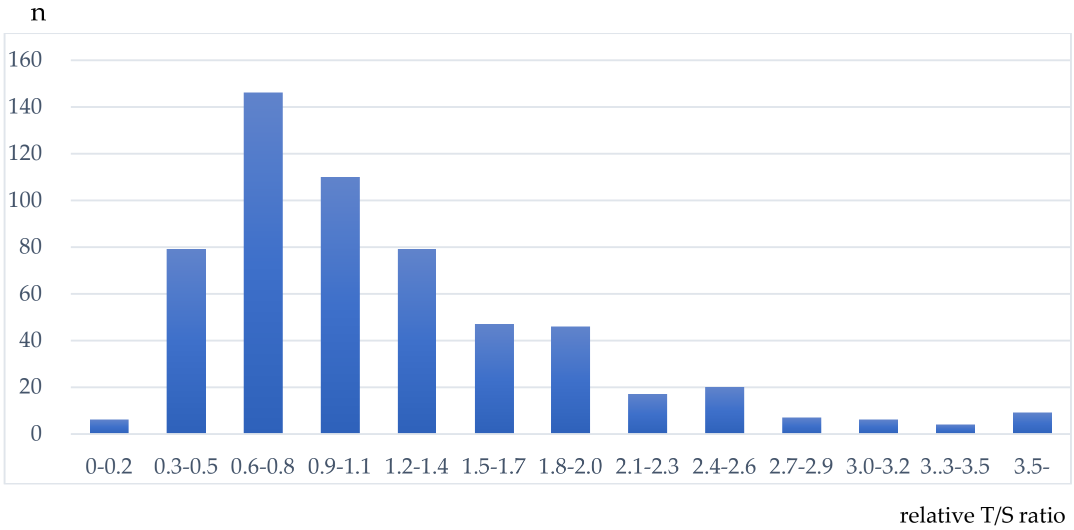

Figure . S2. Distribution of neonatal telomere length. T/S ratio: telomere length to single-copy gene ratio.
